# Resting-State Functional Magnetic Resonance Imaging Networks as a Quantitative Metric for Impact of Neurosurgical Interventions

**DOI:** 10.3389/fnins.2021.665016

**Published:** 2021-10-29

**Authors:** Peter H. Yang, Carl D. Hacker, Bhuvic Patel, Andy G. S. Daniel, Eric C. Leuthardt

**Affiliations:** ^1^Department of Neurological Surgery, Washington University School of Medicine, St. Louis, MO, United States; ^2^Department of Biomedical Engineering, Washington University in St. Louis, St. Louis, MO, United States; ^3^Department of Neuroscience, Washington University School of Medicine, St. Louis, MO, United States; ^4^Center for Innovation in Neuroscience and Technology, Washington University School of Medicine, St. Louis, MO, United States; ^5^Brain Laser Center, Washington University School of Medicine, St. Louis, MO, United States

**Keywords:** functional neuroimaging, humans, laser therapy, magnetic resonance imaging, brain neoplasms/surgery, brain neoplasms/diagnostic imaging

## Abstract

**Objective:** Resting-state functional MRI (rs-fMRI) has been used to evaluate brain network connectivity as a result of intracranial surgery but has not been used to compare different neurosurgical procedures. Laser interstitial thermal therapy (LITT) is an alternative to conventional craniotomy for the treatment of brain lesions such as tumors and epileptogenic foci. While LITT is thought of as minimally invasive, its effect on the functional organization of the brain is still under active investigation and its impact on network changes compared to conventional craniotomy has not yet been explored. We describe a novel computational method for quantifying and comparing the impact of two neurosurgical procedures on brain functional connectivity.

**Methods:** We used a previously described seed-based correlation analysis to generate resting-state network (RSN) correlation matrices, and compared changes in correlation patterns within and across RSNs between LITT and conventional craniotomy for treatment of 24 patients with singular intracranial tumors at our institution between 2014 and 2017. Specifically, we analyzed the differences in patient-specific changes in the within-hemisphere correlation patterns of the contralesional hemisphere.

**Results:** In a post-operative follow-up period up to 2 years within-hemisphere connectivity of the contralesional hemisphere after surgery was more highly correlated to the pre-operative state in LITT patients when compared to craniotomy patients (*P* = 0.0287). Moreover, 4 out of 11 individual RSNs demonstrated significantly higher degrees of correlation between pre-operative and post-operative network connectivity in patients who underwent LITT (all *P* < 0.05).

**Conclusion:** Rs-fMRI may be used as a quantitative metric to determine the impact of different neurosurgical procedures on brain functional connectivity. Global and individual network connectivity in the contralesional hemisphere may be more highly preserved after LITT when compared to craniotomy for the treatment of brain tumors.

## Introduction

Resting-state functional MRI (rs-fMRI) is an imaging technique that has provided significant insight into the functional organization of the brain in both healthy and pathologic states. Functional MRI is based on measurements of changes in T2^∗^ signal that are dependent on the oxy/deoxyhemoglobin ratio; referred to as the blood oxygen level dependent (BOLD) signal. Traditionally, responses to experimental paradigms have been averaged across trials to investigate the topography of specific brain functions. However, even when no cognitive, language, or motor tasks are being performed, networks of brain regions demonstrate synchronous BOLD fluctuations (Biswal et al., 1995; Hacker et al., 2019). Extensive analyses of the correlation patterns of BOLD fluctuations have revealed networks such as the somatosensory, language, visual and auditory networks, among others (Power et al., 2011; Yeo et al., 2011). Importantly, these resting-state networks (RSNs) correspond anatomically to the topography of task-based fMRI activity (Smith et al., 2009; Hacker et al., 2019; Park et al., 2020). In the research setting, rs-fMRI has been used to evaluate network connectivity in disease states such as Parkinson’s and Alzheimer’s disease, providing insight into the impact of these diseases on brain function (Greicius et al., 2004; Hacker et al., 2012). Additionally, clinical outcomes after acute ischemic stroke have been correlated with inter-hemispheric connectivity measured using rs-fMRI. In a study of 13 patients who experienced subcortical stroke, rs-fMRI scans were taken at predetermined time points and compared with those from matched controls. The functional connectivity between the contralesional primary sensorimotor cortex and the bilateral primary sensorimotor cortex in patients who experienced stroke was found to initially decrease then increase over time and correlated with clinical recovery of motor function (Xu et al., 2014). This suggests that functional connectivity and neuroplasticity can be dynamically quantified after neurologic injury using rs-fMRI. Importantly, this demonstrates that the contralateral, non-lesional, hemisphere is useful in measuring changes in RSNs from intracranial lesions. Combined with diffusion tensor imaging for tractography, rs-fMRI is an emerging tool for preoperative neurosurgical planning as well as an intra-operative navigational technique for avoidance of eloquent structures (Dierker et al., 2017; Roland et al., 2017, 2019; Leuthardt et al., 2018).

Post-neurosurgical changes in network connectivity have been evaluated using rs-fMRI in several select applications, including after deep brain stimulation for Parkinson’s disease, corpus callosotomy for epilepsy, and laser ablation of seizure foci (Johnston et al., 2008; Kahan et al., 2014; Boerwinkle et al., 2018). In all instances of studying rs-fMRI in neurosurgical procedures, the surgical intervention was intended to modify neural circuitry and rs-fMRI was used to describe those circuitry changes. However, there has not been a quantitative approach to comparing the functional impact of multiple surgical techniques in treating similar pathologies. With new and more widespread use of minimally invasive procedures such as stereotactic laser interstitial thermal therapy (LITT) to treat intracranial lesions, there is a need for quantifying and comparing the functional impact on brain connectivity to that of conventional open craniotomy.

LITT is a minimally invasive, ablative neurosurgical procedure that has gained significant traction over the past decade. Despite first being described by Bown (1983) the technique has only more recently become viable due to the development of MRI thermometry, which allows for real time monitoring of temperatures distant from the laser probe (De Poorter et al., 1995; Hawasli et al., 2013). The ability to create targeted, conformal thermal lesions in a minimally invasive manner makes LITT an attractive option for treating numerous central nervous system (CNS) lesions. LITT has been described for the treatment of epileptic foci, primary and metastatic brain tumors, and radiation necrosis, with new indications continuing to be developed (Hawasli et al., 2011, 2014; Torres-Reveron et al., 2013; Mohammadi et al., 2014; Kamath et al., 2017; Bijanki et al., 2020; Hafez et al., 2020; Remick et al., 2020). For example, rs-fMRI has been used at our institution during stereotactic LITT procedures both to plan a trajectory that avoids traversing eloquent cortex and to create an ablative lesion that spares eloquent cortex (Salehi et al., 2018). Anecdotally, LITT is presumed to be less disruptive to the overall functional brain connectivity because of the lack of brain retraction and manipulation which occur during open craniotomy, but the details of the differences in functional impact of LITT vs. standard craniotomy have not yet been quantified.

In this work, we demonstrate for the first time the use of a simple yet reproducible quantitative approach using rs-fMRI to illustrate the differences in impact that two surgical approaches have on the functional organization of the brain in patients with singular intra-axial brain tumors. Specifically, we show that the within-hemisphere resting-state Pearson correlation coefficients *contralateral* to the lesion in craniotomy and LITT patients can be directly compared and quantified while overcoming the challenges of tumor mass effect and post-surgical changes and while taking into account individual patient variations.

## Materials and Methods

### Patient Selection

All aspects of this retrospective cohort study were approved by the Human Research and Protection Office Institutional Review Board (IRB) at our institution. Data including patient demographics, surgery types, surgery dates, radiation dates, rs-fMRI accession numbers, and tumor characteristics were retrospectively collected from patients who underwent craniotomy and/or LITT for intra-axial tumors between 2014 and 2017. Informed consent was not obtained due to the retrospective nature of the study. Treatment characteristics included whether patients underwent radiation between scans, prior stereotactic needle biopsy only, LITT, and craniotomy. Tumor characteristics included whether the tumor was intra-axial, type (metastatic or intrinsic glial neoplasm), WHO grade (if applicable), and volume (taken from radiology report). These characteristics were used to match patients between groups. Patients with evidence of bilateral tumor involvement, midline shift, or prior cranial surgery (except stereotactic needle biopsy) were excluded. Patients with post-operative imaging up to 2 years were considered. Statistical analysis of continuous and categorical variables of patient and tumor characteristics were performed using the two-tailed Student’s *t*-test and two-tailed Fisher’s exact test, respectively, to calculate statistical significance (Table 1).

**TABLE 1:** Baseline characteristics of craniotomy and laser interstitial thermal therapy patients.

**Patient characteristics**	**Craniotomy**	**LITT**	***P*-value**
Patients	12	12	–
Mean age	46.7 (23.0–76.0)	48.7 (7.5–68.8)	0.8027
Female	6	5	1.0000
**Tumor characteristics**			
Intra-axial	12	12	–
Metastasis	3	3	0.8948
Intrinsic glial neoplasm	9	9	
WHO grade I/II	5	4	
WHO grade III/IV	4	5	
Mean tumor volume (cm^3^)	14.3713	10.9382	0.6291
**Treatment**			
Radiation between scans	5	2	0.3707
No prior surgery	11	6	0.0686
Prior needle biopsy only	1	6	
**Mean time between surgery and post-operative scan (days)**	235.6667	206.3333	0.9076

*WHO, World Health Organization; LITT, laser interstitial thermal therapy. There was no significant difference between age, gender, tumor etiology, tumor volume, radiation treatment, prior surgery, and time between surgery and post-operative scan. Two tailed Student’s t-test assuming unequal variances for continuous variables. Two-tailed Fisher’s exact test for categorical variables.*

### MRI Image Acquisition

All imaging was performed with a 3T Siemens Trio scanner. Patients were instructed to keep their eyes open and look at a single focus point during image acquisition. All patients remained awake during the scans. Structural imaging included one T1-weighted MP-RAGE [repetition time (TR) = 2,000 ms, echo time (TE) = 2.5 ms, flip angle = 12°, voxel size 1.0 × 1.0 × 1.0 mm] and one T2-weighted turbo-spin echo sequence [TR = 9,000 ms, TE = 115 ms, flip angle = 120°, voxel size 1.0 × 1.0 × 2.5 mm]. Rs-fMRI was acquired using an echo-planar imaging (EPI) sequence sensitive to BOLD contrast (TR = 2,070 ms, TE = 25 ms, flip angle = 90°, voxel size 4.0 × 4.0 × 4.0 mm). Two runs of 200 frames each (∼14 min total) were acquired in each subject.

### Resting-State Functional Connectivity Preprocessing

fMRI preprocessing followed previously published preprocessing methods to ensure that artifacts were removed and that data was normalized (Shulman et al., 2010; Power et al., 2014; Leuthardt et al., 2018; Seitzman et al., 2020). This step corrected for head movement, normalized signal intensity across scans, and performed atlas transformation. Volumetric BOLD time series were registered to an isotropic 3 mm atlas space. In addition, spatial smoothing and voxel-wise temporal smoothing was performed for each run. Temporal frequencies < 0.1 Hz were retained. Time series sampled from regions of cerebrospinal fluid and white matter were used to reduce erroneous variations by regression of nuisance waveforms derived from motion correction. The whole-brain signal was removed as a nuisance regressor. Finally, frame censoring was performed. Volumetric frames in which the root-mean-square of voxel intensities within brain regions changed significantly compared to the previous frame (>0.5% of root-mean-square voxel intensities), the frame was censored to minimize the impact of head motion on functional connectivity computations. Scans were excluded if they failed preprocessing due to significant.

### Resting-State Functional Connectivity Analysis

**FIGURE 1:**
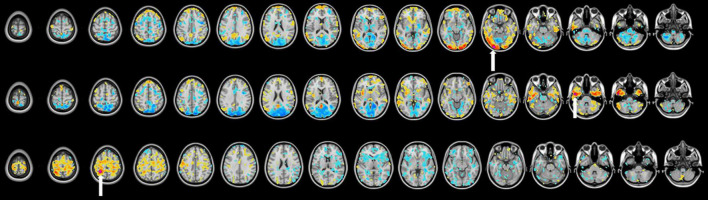
Seed-based correlation map of a single patient with 3 representative ROIs (regions of interest) belonging to different RSNs (resting-state networks) projected onto a reference T1-weighted MRI. The purple dot (labeled with a white arrow) represents the seed ROI and warmer-colored regions represent positive correlation. Cooler colors represent negative correlation. 150 contralesional ROIs were isolated for analysis.

A publicly available set of 300 cortical, subcortical, and cerebellar ROIs,^1^ described in detail by Seitzman et al. (2020), was used in this study (Seitzman et al., 2020). This link also included a list of coordinates and consensus functional network labels. These ROIs and their corresponding network assignments were determined using a combination of seed-based correlation analysis and network community detection. The seed-based correlation analysis was performed to examine the spatial and temporal relationships between ROIs (Figure 1). In short, after image acquisition and preprocessing, the BOLD time series for each seed ROI were calculated as a correlation with all other ROIs in the brain, resulting in a Pearson’s r matrix 300 × 300 in size. Community detection was used to determine the functional network membership of each ROI using the Infomap algorithm (Power et al., 2011). Thus, the 300 ROIs were assigned to one of 14 functional network communities: default mode, frontoparietal, cinguloopercular, salience, dorsal attention, ventral attention, visual, auditory, somatomotor dorsal, somatomotor lateral, parieto-medial, striatal orbitofrontal amygdalar, medial temporal lobe, and unlabeled. Because the latter 3 networks were less well-defined in literature, we selected to examine the former 11 well-defined networks for our study. A system called the Translational Imaging Portal (TIP), a customized version of the Extensible Neuroimaging Archive Toolkit (XNAT) imaging informatics software platform, was used to perform preprocessing and functional connectivity analysis (Leuthardt et al., 2018).

### Analysis of Resting-State Functional Connectivity Resultant From Surgery

Given that postsurgical changes such as edema, pneumocephalus, hemorrhage, craniotomy plating systems, and other sources of artifact can produce noise that can significantly alter the BOLD signal, the entire hemisphere ipsilateral to the tumor was excluded in the correlation analysis. In other words, hemispheric and individual RSN correlation analysis was performed exclusively using within-hemisphere correlations *contralateral* to the lesion The 300 × 300 Pearson’s *r* matrix of the whole brain was therefore truncated into a 150 × 150 hemispheric matrix prior to any statistical analysis.

To control for the variability in tumor size and location between patients, hemispheric and individual RSN correlation analysis was performed using a pair of pre-operative and post-operative scans from the same patient. Specifically, the 2-dimensional correlation coefficient *c* was calculated between any pre-operative matrix (*A*) and the corresponding post-operative matrix (*B*), for each patient (shown below):


$$c_{x,y}=\frac{\sum_{m}\sum_{n}\left(A_{m\,n}-\bar{A}\right)\,\left(B_{m\,n}-\bar{B}\right)}{\sqrt{\left(\sum_{m}\sum_{n}{\left(A_{m\,n}-\bar{A}\right)}^{2}\right)\,\left(\sum_{m}\sum_{n}{\left(B_{m\,n}-\bar{B}\right)}^{2}\right)}}$$

Where $\bar{A}$ = mean of all values in matrix *A* and $\bar{B}$ = mean of all values in matrix *B*, *m* and *n* = column and row indices, *x* = patient number y = name of network represented by *A* and *B*

For example, the correlation coefficient between the pre-operative (*A*) and post-operative (*B*) matrices representing the somatodorsal network (SDN) for patient 1 in the LITT group is calculated by the following:


$$c_{L\,I\,T\,T\,\_\,1,S\,D\,N}=\frac{\sum_{m}\sum_{n}\left(A_{m\,n}-\bar{A}\right)\,\left(B_{m\,n}-\bar{B}\right)}{\sqrt{\left(\sum_{m}\sum_{n}{\left(A_{m\,n}-\bar{A}\right)}^{2}\right)\,\left(\sum_{m}\sum_{n}{\left(B_{m\,n}-\bar{B}\right)}^{2}\right)}}$$

Therefore, every comparison of interest between a pre-operative and post-operative network (matrix) *y* for an individual patient *x* can be represented by its correlation coefficient *c_*x*__,y_*. To quantify the changes for any given network *y* within each surgery group of N patients, the group-wise correlation coefficient *G*_*y*_ was calculated by taking the mean of all *c*_*x,y*_ in each surgery group (i.e., *c_*LITT_1*__, y_, c_*LITT_2, y*_, …c_*LITT_N, y*_)*.

Because craniotomy is the standard of care for tumor treatment, we tested the hypothesis that the functional networks in patients who underwent LITT were not more disrupted than those in patients who underwent craniotomy. When multiple post-operative rs-fMRI scans for a single patient were available, the most recent scan and therefore the longest time interval between scans was used to favor analysis of long-term network changes. The one-tailed Student’s *t*-test, assuming unequal variances, was used to calculate statistical significance. A *P*-value less than 0.05 was taken to indicate significant difference. All matrix comparisons were calculated using MATLAB software (MATLAB and Statistics Toolbox Release 2016b, The MathWorks, Inc., Natick, Massachusetts, United States).

## Results

### Patient and Tumor Characteristics

12 craniotomy patients and 12 LITT patients, each with one pre-operative and one post-operative scan, were included in this study. Patient demographics are shown in Table 1. The mean age and gender distributions were similar between groups. All tumors were intra-axial. Most tumors were intrinsic glial neoplasms and was no statistical difference between metastases and intrinsic glial neoplasms between groups. Patients who underwent LITT trended toward being likely to have had a prior needle biopsy compared to craniotomy patients. Patients who underwent craniotomy trended toward having larger tumor volumes but this was not statistically significant difference between the two groups. There was no significant difference between groups for the number of patients who underwent radiation between scans and the time intervals between surgery and post-operative scan.

### Tumor Location

**FIGURE 2:**
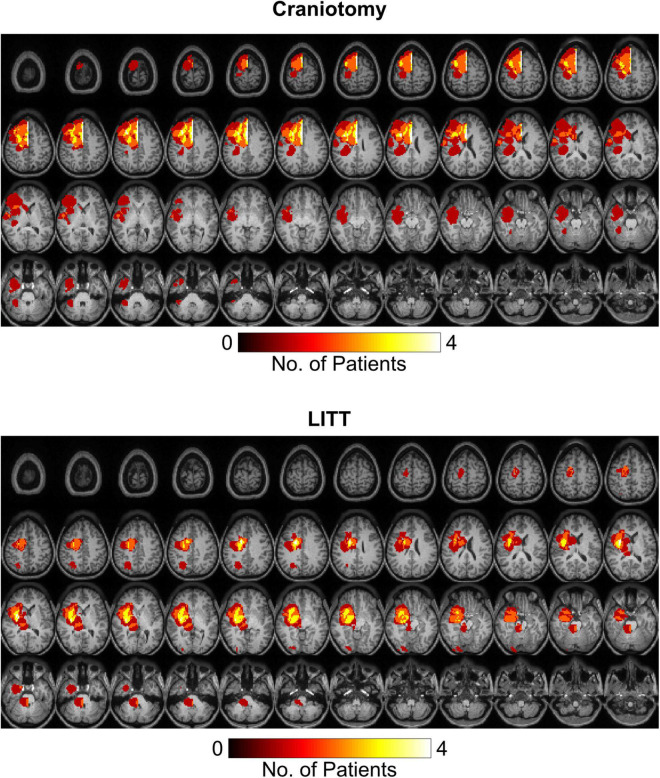
Heat maps representing tumor locations in the craniotomy and LITT (laser interstitial thermal therapy) treatment groups. All tumors were mapped to the right hemisphere for a clearer illustration of tumor location. T1-weighted post-contrast images were used for tumors with contrast enhancement while FLAIR (fluid attenuated inversion recovery) hyperintensity was used for tumors without contrast enhancement.

Tumor segmentation was performed using the T1-weighted post gadolinium contrast images. If the tumors did not have contrast enhancement, the region with FLAIR (fluid attenuated inversion recovery) hyperintensity was used. Tumor frequency maps illustrate the spatial distribution of tumor locations across patients (Figure 2). Tumors in the left hemisphere were reflected onto the right hemisphere for ease of visual comparison. Supplementary Figure 1 delineates all regions with tumor-related changes (i.e., contrast enhancement plus surrounding FLAIR hyperintensity).

### Network Analysis

**FIGURE 3:**
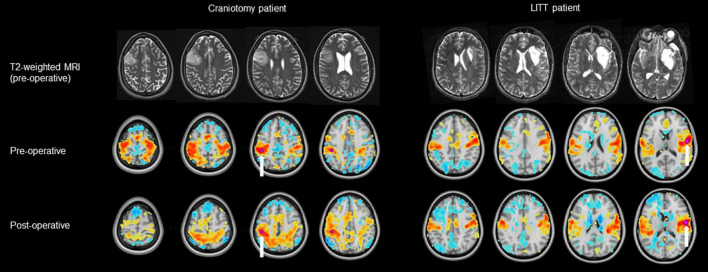
Examples of a pre-operative and post-operative RSN (resting-state network) using a tumor-adjacent seed ROI (region of interest, labeled with a white arrow) on the contralesional hemisphere. The LITT (laser interstitial thermal therapy) patient (right) had a greater degree of correlation between the pre-operative and post-operative RSN than did the craniotomy patient (left). Note: clinical images use anatomic orientation while RSN projections use neurologic orientation.

**FIGURE 4:**
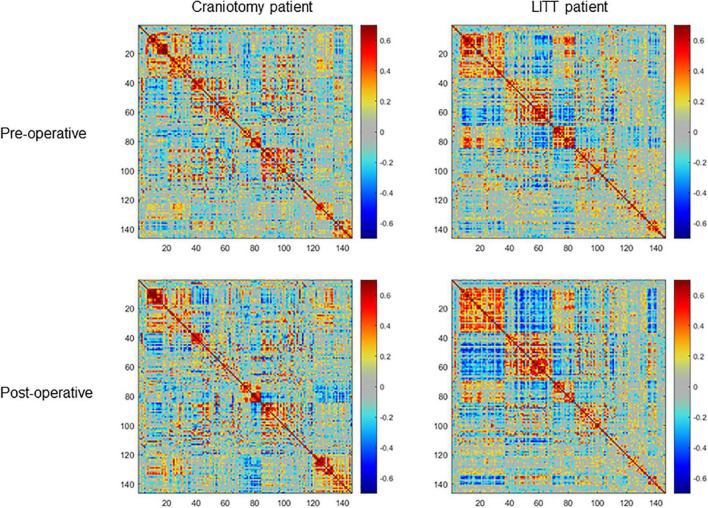
Examples of pre-operative and post-operative within-hemisphere correlation matrices of the contralesional hemisphere (ROIs (regions of interest) = 150) of a single craniotomy patient (left) and a single LITT (laser interstitial thermal therapy) patient (right). The LITT patient had a greater degree of correlation between the pre-operative and post-operative matrices than did the craniotomy patient as determined by the 2-dimensional correlation coefficient.

ROIs for each patient were grouped into a total of 11 RSNs and one 150 × 150 hemispheric correlation matrix then compared between craniotomy patients and LITT patients (Figures 3, 4). The functional network names, abbreviations, and the number of ROIs comprising each network are shown in Table 2.

**TABLE 2:** Resting-state networks and group-wise two-dimensional correlation coefficients between pre-operative and post-operative scans.

**Abbreviation**	**Resting-state network**	**ROIs**	**Craniotomy**	**LITT**	***P*-value**
SDN	Somatomotor Dorsal	17	0.667	0.741	**0.0343**
SLN	Somatomotor Lateral	3	0.969	0.965	0.4094
CON	Cingulo-Opercular	8	0.873	0.893	0.2263
AN	Auditory	6	0.816	0.818	0.4558
DMN	Default Mode	31	0.652	0.694	0.1308
PMN	Parieto-Medial	3	0.976	0.971	0.3445
VN	Visual	19	0.698	0.746	0.1164
FPN	Fronto-Parietal	15	0.710	0.825	**0.0001**
SN	Salience	8	0.792	0.880	**0.0081**
VAN	Ventral Attention	5	0.879	0.916	0.1360
DAN	Dorsal Attention	7	0.817	0.897	**0.0032**

*One-tailed Student’s t-test demonstrates a higher degree of correlation between the pre-operative and post-operative Somatomotor Dorsal, Fronto-Parietal, Salience, and Dorsal Attention resting-state networks in patients who underwent laser interstitial thermal therapy. ROIs, regions of interest; LITT, laser interstitial thermal therapy. Bold numbers indicate *P* < 0.05.*

**FIGURE 5:**
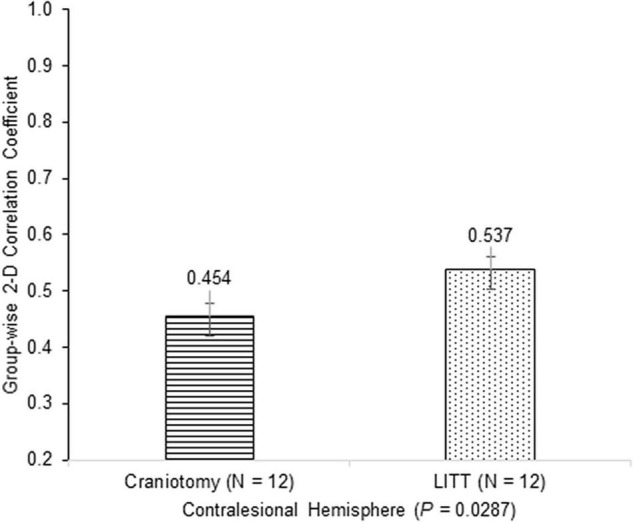
Group-wise 2-dimensional correlation coefficients between pre-operative and post-operative hemispheres between patients who underwent craniotomy and LITT (laser interstitial thermal therapy). There was a greater degree of correlation in patients who underwent LITT (one-tailed Student’s *t*-test, assuming unequal variances).

**FIGURE 6:**
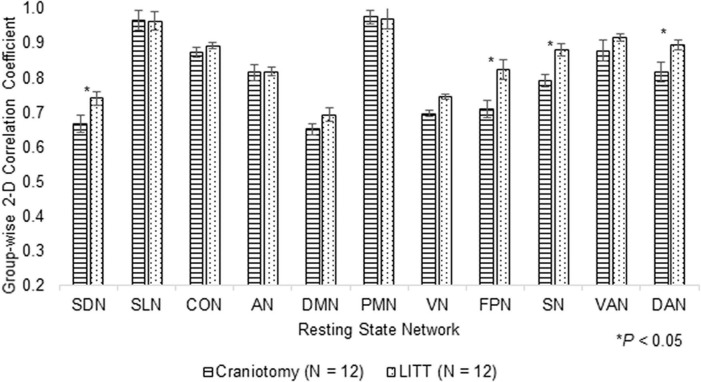
Group-wise 2-dimensional correlation coefficients between pre-operative and post-operative RSNs (resting-state networks) between patients who underwent craniotomy and LITT (laser interstitial thermal therapy). 4 RSNs among LITT patients showed a higher degree of correlation between scans than among craniotomy patients (one-tailed Student’s *t*-test, assuming unequal variances).

Histograms were generated to illustrate the group-wise 2-dimensional correlation coefficients of the pre-operative and post-operative contralesional hemispheres (Figure 5) and 11 RSNs (Figure 6) among patients who underwent craniotomy and those who underwent LITT. A correlation coefficient closer to 1 implies a network that is more similar to that of the pre-operative state. In our primary analysis, we found that the contralesional hemisphere showed a significantly higher degree of correlation between pre-operative and post-operative connectivity in the patients who underwent LITT compared to those who underwent craniotomy (Figure 5, *P* = 0.0287). In our secondary exploratory analysis, there was a higher degree of correlation in the connectivity of 4 networks (Somatomotor Dorsal, Fronto-Parietal, Salience, and Dorsal Attention) in patients who underwent LITT (Table 2, Figure 6). In majority of the remaining 7 comparisons, the RSNs in the LITT patients trended toward a greater degree of correlation compared to the craniotomy group.

## Discussion

LITT has been shown to be safe and effective in the treatment of intracranial lesions (Shah et al., 2019; Kim et al., 2020). We have previously reported the outcomes and safety profile in using LITT as a frontline treatment for glioblastomas, metastases, low grade gliomas, epileptic foci, and radiation necrosis (Kamath et al., 2017, 2019; Salehi et al., 2018). While the thermal effects on tissues within the ablation target and the surrounding brain have been characterized, the impact of LITT on intrinsic functional organization of the brain, and specifically when compared to craniotomy, had not yet been explored (Mohammadi and Schroeder, 2014). Here we pilot a novel approach to quantify and compare the hemispheric and individual RSN changes from two surgical approaches while taking into account tumor mass effect, post-surgical changes, and individual patient variability. We found that relative to open craniotomy for tumor resection, LITT may have less of an impact on overall functional architecture, as measured by rs-fMRI connectivity. We found that in the follow-up period up to 2 years (average approximately 6 months) after surgery, the contralesional hemisphere and 4 RSNs showed a statistically significant difference in patients who underwent craniotomy vs. those who underwent LITT. Using this computational method, we found that the overall post-surgical brain functional connectivity in patients who underwent LITT was more similar to the pre-operative state than in patients who underwent craniotomy. These findings may not only provide evidence for the assumption that LITT may disrupt brain functional connectivity less than craniotomy, but that this analytical approach using rs-fMRI may be a useful objective tool for quantifying and comparing the effects that different neurosurgical interventions can have on the functional organization within a patient’s brain.

Based on prior literature, it is reasonable to believe that rs-fMRI changes are clinically meaningful. In a longitudinal study of patients with Parkinson’s disease, for example, Olde Dubbelink et al. (2014) found that FC changes were more pronounced in certain brain regions that corresponded to clinical measures of cognitive decline over time, which was independent from aging effects from the control group. Moreover, FC changes using rs-fMRI may also reflect the effect of therapeutic intervention. A randomized trial of cognitive rehabilitation in Parkinson’s disease found that the intervention group showed increased FC between the bilateral dorsolateral prefrontal cortex and the left inferior temporal lobe, which correlated with measures of executive function. These effects in both FC and cognitive function were preserved at 18-month follow-up (Díez-Cirarda et al., 2018). These findings suggest that FC may correlate with neurocognitive status and serve as a diagnostic tool to monitor clinical change over time. As the overall practice of LITT continues to grow, it is important to understand the neurophysiological and neurocognitive consequences of this technology. The extent of network disruption in LITT as it compares to open resection can have implications not only on recovery after surgery, but potentially on survival. Preservation of motor and speech function after surgery have been show to predict overall survival after glioblastoma resections (McGirt et al., 2009; Gulati et al., 2011).

In considering rs-fMRI and network connectivity as it relates to the intervention, several variables as it relates to surgery were taken into consideration to best enable a fair comparison between the two interventions. First, laser ablation alters blood brain barrier (BBB) permeability which may have implications on glial-neuronal interactions that drive the BOLD signal. Our previous work has shown evidence that the BBB may be disrupted by hyperthermia, that peaks at 1–2 weeks after surgery and resolves by 4–6 weeks (Leuthardt et al., 2016). To reduce the potential effect of BBB permeability on BOLD signaling that might have unintended impact on detecting differences between RSNs, we selected scans from the most recent timepoint in order to minimize the impact of these changes when multiple post-operative scans were available. Second, the treated hemisphere is subject to a number of confounding variables that could impact network organization. The mass effect of the intracranial lesion on surrounding structures, peritumor edema, surgical plating systems, and post-surgical changes have posed challenges when studying RSNs. Prior studies investigating the changes in RSNs when there is brain asymmetry have used comparison methods using the *contralesional* hemisphere only (Park et al., 2011; Liu et al., 2020). Here, we used a similar approach, isolating and analyzing the within-hemisphere connectivity of RSNs of the contralesional hemisphere. This analytical technique makes the assumption that the intracranial pathology impacts the FC of the contralesional hemisphere, and importantly, this effect is indirect and nontrivial. This assumption is advantageous for the ease of mathematical analysis, but also indicates that local tissue disruption may have secondary global effects.

This study has several limitations including its retrospective nature, small patient cohort, and the lack of correlations to neurocognitive outcomes that might have clinical implications as mentioned above. Direct comparison of resting-state changes between craniotomy and LITT assumes similar baseline tumor characteristics, but in clinical practice there are likely inherent differences between these treatment groups such as tumor location and tumor volume which factor into the decision of which surgery is pursued. Therefore, the main limitation of this study is the inherent mismatch between treatment groups. However, this is the first study to use a quantitative approach to comparing the effect of open and minimally invasive tumor surgery on functional connectivity and to show that LITT may be less disruptive to intrinsic networks than conventional craniotomy using rs-fMRI as a quantitative metric. We demonstrated that analyzing the within-hemisphere connectivity within the contralesional hemisphere can be a useful strategy when comparing RSNs in the setting of mass effect, and patients serving as their own control can help reduce the individual variability when comparing patient groups. Future studies are warranted and include validating these results using patient cohorts matched by tumor location, volume, and type, quantifying the time course of RSN recovery after surgery, and correlating these changes to neurocognitive outcomes.

## Conclusion

The purpose of this study was to use a novel method to quantify and compare changes in functional network connectivity in patients who underwent craniotomy and LITT for the treatment of intra-axial brain tumors. Global hemispheric connectivity and 4 individual RSNs were more well-preserved in patients who underwent LITT than those who underwent craniotomy in a follow-up period up to 2 years. Larger studies are warranted to validate these results and to determine their clinical implications.

## Data Availability Statement

The raw data supporting the conclusions of this article will be made available by the authors, without undue reservation.

## Ethics Statement

The studies involving human participants were reviewed and approved by the Washington University in St. Louis. Written informed consent from the participants’ legal guardian/next of kin was not required to participate in this study in accordance with the national legislation and the institutional requirements.

## Author Contributions

PY, BP, and EL contributed to hypothesis generation. PY and BP performed data collection and primary data analysis. PY was responsible for coding, statistical analysis, and manuscript writing. BP, CH, and EL performed manuscript editing. CH and AD assisted with secondary data analysis and visualization. All authors contributed to the article and approved the submitted version.

## Conflict of Interest

EL is a consultant for Monteris Medical, E15, Acera, Alcyone, Intellectual Ventures, Medtronic Inc., Neurolutions, Osteovantage, Pear Therapeutics, Inc., Sante Ventures, and Microbot, owns equity in Neurolutions, General Sensing, Osteovantage, Pear Therapeutics, Face to Face Biometrics, Immunovalent, Caeli Vascular, Acera, Sora Neuroscience, Inner Cosmos, and Kinetrix, and is involved with licensing intellectual property in Neurolutions, Osteovantage, Caeli Vascular, Cerovations, and Intellectual Ventures. Washington University owns equity in Neurolutions. CH owns equity in Sora Neuroscience. The remaining authors declare that the research was conducted in the absence of any commercial or financial relationships that could be construed as a potential conflict of interest.

## Publisher’s Note

All claims expressed in this article are solely those of the authors and do not necessarily represent those of their affiliated organizations, or those of the publisher, the editors and the reviewers. Any product that may be evaluated in this article, or claim that may be made by its manufacturer, is not guaranteed or endorsed by the publisher.

## Footnotes

1
https://greenelab.ucsd.edu/data_software

## Supplementary Material

Supplementary Figure 1 Heat maps representing tumor-affected regions in the craniotomy and LITT (laser interstitial thermal therapy) treatment groups. T1-weighted post-contrast images and surrounding FLAIR hyperintensity were used for tumors with contrast enhancement. FLAIR hyperintensity was used for tumors without contrast enhancement.
