# Supplementary figures and images for: Resting-State Functional Magnetic Resonance Imaging Networks as a Quantitative Metric for Impact of Neurosurgical Interventions
Source: Front Neurosci. 2021 Oct 29;15:665016. doi: 10.3389/fnins.2021.665016 (PMC8585791; doi:10.3389/fnins.2021.665016)

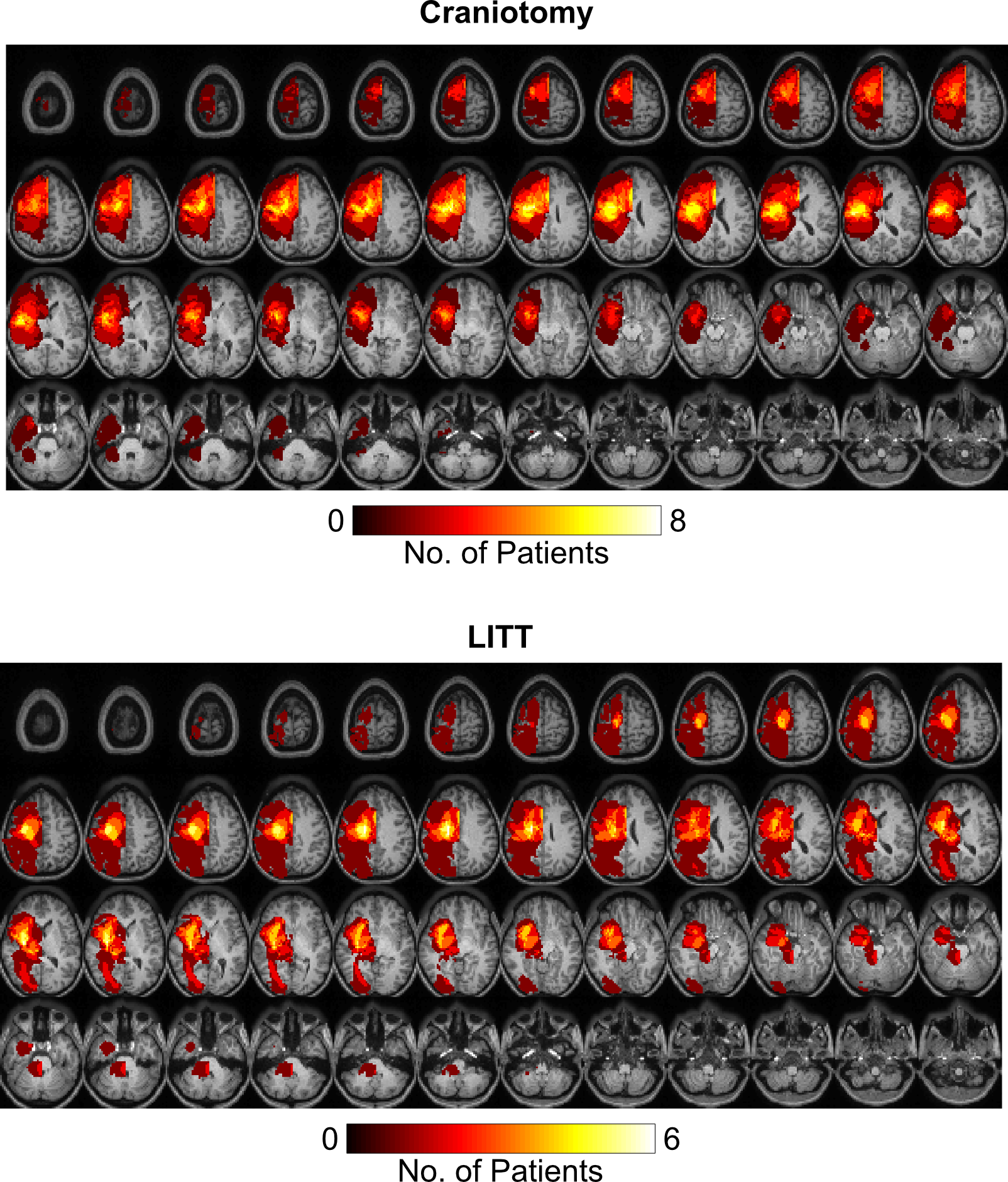

Supplement: Supplementary Figure 1 — Heat maps representing tumor-affected regions in the craniotomy and LITT (laser interstitial thermal therapy) treatment groups. T1-weighted post-contrast images and surrounding FLAIR hyperintensity were used for tumors with contrast enhancement. FLAIR hyperintensity was used for tumors without contrast enhancement. [file Image_1.TIFF]
